# Code of Ethics of the Massachusetts State Medical Society

**Published:** 1880-04

**Authors:** 


THE CODE OF ETHICS OF THE MASSACHUSETTS STATE MEDICAL SOCIETY.—Until recently the Massachusetts State Medical Society had no formal code of ethics. Some of the more important practical items of the general code were incorporated into the by-laws of the society. During the last year, however, the propriety of adopting a more full and distinctly expressed code of ethical rules, has engaged much of the attention of its members. The discussion was ended at a recent meeting of the society by the adoption of a concise set of ethical rules, specially adapted to the organization of the society, and in several places referring to particular sections of its by-laws.

As the Massachusetts Medical Society is one of the oldest and most respectable medical society organizations in this country, we are glad she has formally adopted a code of ethics, thereby acknowledging the propriety and value of some clearly expressed written rules for the guidance of members of the profession in their relations to each other, and to their patients. It has been quite the custom of some occupying prominent positions in the profession, to sneer at the idea of a written code of ethics, claiming that it was derogatory to the dignity of an honorable profession to acknowledge anything beyond the *golden rule.*

Such, however, take a very narrow view of the subject, apparently seeing in the code, nothing but a system of penal rules. They do not seem to comprehend the fact that a written code is not only designed as a rule of conduct, but an exposition of the true relations of the members of the profession to each other, to their patients and to the community, by the reading of which many honest, plodding, well-meaning members, are enlightened and their views of duty made more clear and elevated; many less scrupulous are restrained; and in cases of doubt and disagreement, all have a tangible, uniform standard for their guidance. Then we say again that we are pleased with the action of the Massachusets State Medical Society, more especially as the code it has adopted, so far as it goes, differs in no important particular from the provisions concerning the same topics, in the National Code, so generally adopted by the medical societies in this country. To satisfy the curiosity of our readers, we give below the Massachusetts Code as published in the Boston *Medical and Surgical Journal:*

(Adopted by the Councilors, February 4, 1880).

Object of a code of ethics. The Massachusetts Medical Society is designed to secure to the public a body of well educated and otherwise trusty physicians. Its code of ethics is intended to furnish certain principles and rules of action for their guidance and convenience.

I. The relation of the physician to medical science. A physician should lend his influence to encourage sound medical education, and to uphold in the community correct views of the powers and the limitations of medical science and art.

II. The relation of the physician to medical business. The professional success of a practitioner depends upon qualities connected with his moral character, his scientific attainments, and also his industry and business talent. But the relation of practitioners of medicine to families and households is not like that of tradesmen to their customers. The kind of competition which might be considered honorable in business, cannot exist between physicians without diminishing their usefulness and lowering the standard of the medical profession. (See IV, Sec. 1; V, Sec. 1).

III. The relation of the physician to his patients. The first duty of the practicing physician is to his patient, who has a right to expect that his disease shall be thoroughly investigated and skillfully treated, with charitable consideration for his mental peculiarities or infirmities, and in a relation strictly confidential.

1. The physician should not make unnecessary visits. He should neither permit needless apprehension nor fail to give seasonable notice of danger.

IV. The relation of the physician to other practitioners and to their patients. In his relations with another practitioner and his patients, a physician should be governed by strict rules of honor and courtesy. His conduct should be such as, if universally imitated, would insure the mutual confidence of all medical practitioners.

The foregoing rule should be a sufficient guide of action. Some of the following contingencies will illustrate its application:

1. A physician should take no step with a view directly or indirectly to divert to himself the patients or practice of another physician.

2. If formally requested to assume charge of a patient or family, usually attended by another physician, he should consent to do so only after notifying the latter, unless the case be one of pressing necessity.

3. If a physician is called to a patient during the temporary absence or illness of the usual physician, or in the case of accident or other emergency, he should direct that the former be sent for as soon as he is able to take charge of the case, and should then relinquish it to him. It is generally agreed that, among several physicians thus called, he who first arrives shall act, unless the family designate another.

4. A communication from the temporary to the usual physician, in the absence of the latter, should be written and sealed, and not simply verbal.

V. The relation of the physician to quackery. In every community there are minds naturally inclined to quackery, which has flourished in every age. It grows by being noticed, and thrives best under opposition. It is commonly unwise to employ argument against it. But a physician should lend his influence to establish a distinct line between the regular practice of medicine and the practice of quackery, and should avoid any act which might tend to weaken such a distinction either in the professional or in the public mind.

1. Thus, he should not consult with an irregular practitioner (*see By-Laws*,) nor countenance the use of secret remedies, nor be interested in medical trademark preparations; nor give certificates recommending mineral waters, patents, or medical preparations, or the like; nor give a commission to an apothecary, nor receive one from him; nor advertise himself or his practice in public print; nor publicly advertise advice or medicines to the poor, etc.

VI. Consultations should be encouraged in cases of unusual responsibility or doubt.

A consultation is called for the benefit of the patient, and to give him the advantage of collective skill. Should there be a difference of opinion, discussion should be temperate and always confidential.

A consulting physician should be careful to say or do nothing to impair the confidence of the patient or his family in the attending physician.

1. See, for guidance of a consultant, IV, Secs. 1, 2, 3, 4.

2. At a consultation punctuality is important; and non-arrival within fifteen minutes after the appointed time should be interpreted as non-attendance.

3. For the advantage of the patient and for economy of time, it is well in a consultation to observe a certian order of business. The following has been found convenient:

The attending physician, having stated in general terms the nature of the case, may then call, in turn, upon each consultant, if there be more than one, to examine the patient,—the usual order being that of seniority. No consultant should make an examination or inquiry out of turn. On retiring, the attending physician may invite, in the usual order, the opinion of each consultant, who should not be interrupted while giving it; after which he may add his own. In conclusion, a course of action may be agreed on, or the attending physician may be left to act at his own discretion.

VII. Fees. A fee-table has a local application, and is designed to indicate a fair or average amount due for services. But if the patient fully understands it beforehand, a physician is at liberty to place any value he sees fit upon his services. It is then at the patient's option to decline them or to pay the price. A physician should be considerate of the poor.

1. A patient in moderate circumstances should not be called upon to pay a fee unusually large for the service rendered, without a previous explicit understanding. A physician, if able, should offer to pay the medical attendant of himself or of his family. Unless by special agreement, a physician attending or acting for another should receive the fees. Among obstetricians a rule obtains that the interval between the birth of the child and the placenta halves the service and the fee. A fee should be charged for a medical certificate or paper of value to the applicant, connected with, for example, absence or exemption, life insurance, pension papers, etc., except the usual certificates of vaccination and death.

VIII. Seniority. Seniority applies rather to duration of practice at the place in question than to age.

